# Corrigendum

**DOI:** 10.1111/mpp.12845

**Published:** 2019-07-19

In Fernández‐Calvino *et al*. (2016), published in *Mol. Plant Pathol*. 17, 3‐15, the second author, Irene Guzmán‐Benito, had requested to add another affiliation.

The author byline and affiliations should then read as follows:

LOURDES FERNÁNDEZ‐CALVINO^1,^†, IRENE GUZMÁN‐BENITO^1,2^, FRANCISCO J. DEL TORO^1^, LIVIA DONAIRE^1^, ANA B. CASTRO‐SANZ^1,^†, VIRGINIA RUÍZ‐FERRER^1^ AND CÉSAR LLAVE^1,^*

^1^ *Department of Environmental Biology, Centro de Investigaciones Biológicas, CSIC, Ramiro de Maeztu 9, 28040 Madrid, Spain*

^2^ *Doctorado en Biotecnología y Recursos Genéticos de Plantas y Microorganismos Asociados, ETSI Agronómica, Alimentaria y de Biosistemas, Universidad Politécnica de Madrid, 28040‐Madrid, Spain*

The published version of the article has been corrected online.

The authors apologize for any inconvenience it may have caused.

**REFERENCE**

**Fernández‐Calvino, L., Guzmán‐Benito, I., del Toro, F.J., Donaire, L., Castro‐Sanz, A.B., Ruíz‐Ferrer, V. and Llave, C.** (2016) Activation of senescence‐associated *Dark‐inducible (DIN)* genes during infection contributes to enhanced susceptibility to plant viruses. *Mol. Plant Pathol.* **17**, 3–15.

